# Lead Induced Hepato-renal Damage in Male Albino Rats and Effects of Activated Charcoal

**DOI:** 10.3389/fphar.2017.00107

**Published:** 2017-03-14

**Authors:** Samuel J. Offor, Herbert O. C. Mbagwu, Orish E. Orisakwe

**Affiliations:** ^1^Department of Pharmacology and Toxicology, Faculty of Pharmacy, University of UyoUyo, Nigeria; ^2^Department of Experimental Pharmacology and Toxicology, Faculty of Pharmacy, University of Port HarcourtPort Harcourt, Nigeria

**Keywords:** chronic lead toxicity, activated charcoal, hepato-renal damage, biomarkers, public health

## Abstract

Lead is a multi-organ toxicant implicated in various cancers, diseases of the hepatic, renal, and reproductive systems etc. In search of cheap and readily available antidote this study has investigated the role of activated charcoal in chronic lead exposure in albino rats. Eighteen mature male albino rats were used, divided into three groups of six rats per group. Group 1 (control rats) received deionised water (10 ml/kg), group 2 was given lead acetate solution 60 mg/kg and group 3 rats were given lead acetate (60 mg/kg) followed by Activated charcoal, AC (1000 mg/kg) by oral gavage daily for 28 days. Rats in group 2 showed significant increases in serum Aspartate aminotransferase, Alkaline phosphatase, Alanine aminotransferase, urea, bilirubin, total cholesterol, triglycerides, Low Density Lipoprotein, Very Low Density Lipoproteins, Total White Blood Cell Counts, Malondialdehyde, Interleukin-6, and decreases in Packed Cell Volume, hemoglobin concentration, Red blood cell count, total proteins, albumins, superoxide dismutase, glutathione peroxidase and total glutathione. Co-administration of AC significantly decreased these biomarkers with the exception of the sperm parameters. Histopathology of liver and kidney also confirmed the protective effective of AC against lead induced hepato-renal damage. AC may be beneficial in chronic lead induced liver and kidney damage.

## Introduction

Lead constitute an integral source of poisoning to the ecosystem. It primarily affects the central nervous system, hematopoietic, hepatic and renal system, producing serious disorders (Kalia and Flora, 2005). Lead exposure also causes anemia, immunotoxicity and toxicity to the reproductive organs (WHO, 2016). Excessive dietary intake of lead has been linked with cancers of stomach, small intestine, large intestine, ovary, kidney, lungs, myeloma, all lymphomas, and all leukemia (Reddy et al., 2004). Studies in general populations have identified a positive association of lead exposure with coronary artery disease (CAD), stroke mortality, and peripheral arterial disease (Lustberg and Silbergel, 2002; Prozialeck et al., 2008). Lead has also been reported of causing impairment of the quality of semen in the male reproductive system (Eibensteiner et al., 2005; Telisman et al., 2007). There is no known safe blood lead concentration. Even blood lead concentration as low as 5 μg/dL, once thought to be a safe level, may result in decreased intelligence in children, behavioral difficulties and learning problems (WHO, 2016).

Developing nations are particularly of high risk to lead poisoning and carry the highest burden of this hazard. In Nigeria, a suspected case of lead poisoning which occurred at *Unguwan Magiro* and *Unguwan Kawo* communities in Rafi Local Government Area of Niger state in which 48 cases mostly children, (with BLL between 171.5–224 μg/dL) including 14 deaths were reported in May 15, 2015 (WHO, 2015). In March, 2010, Medecins Sans Frontieres, MSF/Doctors without Borders, an international, independent, medical humanitarian organization was alerted to a high number of child fatalities in Zamfara state, northern Nigeria. An estimated 400 children died. Laboratory testing later confirmed high levels of lead in the blood of the surviving children. The root cause of the lead poisoning crisis was unsafe mining and ore processing (Medecins Sans Frontieres [MSF], 2012). Artisanal gold mining as well as agriculture are the predominant occupations in the affected communities. Lead poisoning from Lead-acid battery recycling was also reported in Dakar, Senegal (WHO, 2010). The current drug treatment of lead poisoning is Chelation Therapy with drugs such as Dimercaprol, Ethylenediaminetetraacetic acid (EDTA), Succimer and D-penicillamine (Cuprimine) (Kessel and O’Connor, 2001).

Activated charcoal is a light, finely divided, black fluffy powder prepared by pyrolysis of carbonaceous material such as wood, coconut shells, or petroleum and oxidation using steam or air at high temperature (600–900°C) (Orisakwe, 1994; Cooney, 1995; Olson, 2010; Vaziri et al., 2013). It adsorbs a wide range of substances and organisms (Cooney, 1995). According to Cooney (1995), the adsorption of most metals including lead to activated charcoal is poor and consequently it is seldom used in management of lead poisoning. While AC is mainly associated with treatment of poisoning substances, it has other important roles in the treatment of patients with chronic kidney disease which enhances the outcome of renal dialysis (Alkhatib and Al Zailaey, 2015).

Many antidotes are biological products and their cost, methods of production, potential for eliciting immunogenic responses, the time needed to generate them, and stability issues contribute to their limited availability and effectiveness. These factors exacerbate a world-wide challenge for providing treatment (Weisman et al., 2015). In resource poor nations of sub Saharan Africa with rampant lead poisoning, the cost of chelation therapy is considered prohibitive. There is a need therefore to explore readily available and natural antidotes in the management of lead poisoning. Hitherto there is sparse information on the *in vivo* adsorptive capacity of activated charcoal on lead (Cheong and Roh, 2006). The present study seeks to add to the fund of knowledge for the clarification of the usefulness of activated charcoal in the management of the hepato-renal complications of chronic lead exposure since available data so far have focused on acute lead exposure.

## Materials and Methods

### Materials

#### Chemicals

Lead acetate trihydrate (May & Baker, England), Activated Charcoal, AC (Merck KGaA, Darmstadt, Germany). Lead acetate salt was dissolved in deionised water, while AC was dispersed in deionised water to form a suspension.

#### Animal Husbandry

Eighteen mature male albino Wistar rats, weighing 145–170 g obtained from the University of Uyo Animal house, were acclimatized for 2 weeks, maintained under controlled conditions of temperature (23 ± 2°C) and humidity (50 ± 5%) and a 12-h light–dark cycle, were used for the experiment. The animals were housed in sanitized polypropylene cages containing sterile paddy husk as bedding. The bedding of the cages was changed daily and the cages were cleaned as well. They had free access to standard rat pellet diet and water *ad libitum*. The procedures were performed according to the guidelines on the use of animals and approved by the Institutional Animal Ethical Committee of the University of Uyo.

#### Experimental Design

The animals were divided into three groups of six rats per group as follows: The animals were treated as follows;

Group 1 – Normal control received deionised water 10 ml/kg by oral gavage daily for 28 days in addition to standard feed and water.
Group 2 – Lead acetate solution 60 mg/kg by oral gavage daily for 28 days addition to standard feed and water (Cheong and Roh, 2006).
Group 3 – Lead acetate (60 mg/kg) (Cheong and Roh, 2006), followed by Activated charcoal AC (1000 mg/kg) by oral gavage daily for 28 days (American Society for the Prevention of Cruelty to Animals [ASPCA], 2015). In all animals received activated charcoal 90 min after administration of lead (Olson, 2010).

#### Necropsy

Treatments continued for 28 days. Blood was collected by retro orbital sinus puncture (Stone, 1954). Each blood sample was divided into two portions. The first one was mixed well with the anticoagulant, dipotassium EDTA by shaking and used for hematological screening. The second portion (without anticoagulant) was kept at room temperature for 30 min to clot. Afterward, the clotted blood sample was centrifuged at 3,000 rpm for 10 min. The clear serum supernatant was then carefully aspirated and stored in a clean sample bottle for the determination of some biochemical parameters. Rats were sacrificed under ether anesthesia (Okolo et al., 2016) and concussion stunning involving manually applied trauma on the head (AVMA, 2013); the kidney and liver were excised, weighed, rinsed in saline, and preserved in 10% formalin for histopathological study.

#### Hematological Screening

Total White Blood Cell Counts (TWBC), Packed Cell Volume (PCV), Red blood cell (RBC) count, Lymphocytes % (L%) and Neutrophils % (N%) were determined using the Hemocytometer method (Thrall and Weiser, 2002). Hemoglobin (Hb) concentration was determined by the Cyanmethemoglobin method (Higgins et al., 2008).

#### Biochemical Analysis

Total serum bilirubin (Doumas et al., 1973), serum total proteins (Lubran, 1978), serum Albumins (Doumas and Peters, 1997), serum Globulin (calculated by subtracting the quantity of albumins from that of total proteins), serum total cholesterol (Allain et al., 1974), High Density Lipoprotein (HDLs) (Albers et al., 1978), Low Density Lipoprotein (LDL) and Very Low Density Lipoproteins (VLDL) (Friedewald et al., 1972; Warnick et al., 1990), serum triglycerides (Bucolo and David, 1973), serum creatinine (Blass et al., 1974) and serum urea (Fawcett and Scott, 1960) and Searcy et al. (1967) were determined. The following liver enzymes were also assayed: Alanine Aminotransferase (ALT) and Aspartate aminotransferase (AST) (Reitman and Frankel, 1957) and Alkaline phosphatase (ALP) (Babson et al., 1966).

#### Antioxidants Study and Assessment of Lipid Peroxidation/Oxidative Stress

##### Determination of Antioxidant Levels Namely

(i) Superoxide dismutase (SOD) in whole blood using Superoxide Dismutase kit in accordance with manufacturer’s recommended protocols (Fortress Diagnostics Limited, UK).
(ii) Glutathione peroxidase (GSH-PX) in whole blood using Glutathione peroxidase kit in accordance with manufacturer’s recommended protocols (Fortress Diagnostics Limited, UK).
(iii) Plasma Total Glutathione using RayBio^®^ Glutathione Colorimetric Detection Kit in accordance with manufacturer’s recommended protocols (RayBiotech, Inc. USA)
(iv) Measurement of Malondialdehyde (MDA), a prototype of the thiobarbituric reactive substances (TBARS) as a biomarker of lipid peroxidation and oxidative stress using the modified thiobarbituric acid method (Todorova et al., 2005).

#### Determination of Serum Levels of Pro-Inflammatory Cytokines

Serum levels of pro-inflammatory cytokines (Tumor necrosis factor-alpha, TNF-α) and Interleukin-6 (IL-6) were determined using rat ELISA (Enzyme-linked immunosorbent assay) kits in accordance with manufacturer’s recommended protocols (RayBiotech, Inc. USA and Assaypro LLC, USA).

### Statistical Analysis

Results were expressed as mean ± standard deviation, SD. Statistical analysis was carried out with one way analysis of variance (ANOVA) followed by Tukey’s HSD *post hoc* test. Values of *p* < 0.05 were considered to be significant.

## Results

### Effect of Activated Charcoal on Hematological Parameters

Treatment of rats with lead acetate (Group 2) caused significant (*p* < 0.05) decreased in PCV, Hb concentration and RBC count when compared with normal control. These decreased parameters were increased significantly (*p* < 0.05) in group 3 animals which were given Activated charcoal after lead acetate treatment. Rats in group 2 (given lead acetate only) also had significant increase (*p* < 0.05) in total white blood count (WBC) when compared to rats in the normal control group (Group 1), while the total WBC in group 3 animals was significantly (*p* < 0.05) decreased. There was no effect on lymphocyte and neutrophil percentages (**Table 1**).

**Table 1:** Effect of Activated charcoal on the hematological parameters of lead acetate-treated male albino Wistar rats.

Treatment	Packed cell volume, PCV (%)	Hemoglobin, Hb concentration (g/dl)	Red blood cell count, RBC (10^6^/μL)	Total WBC count (10^3^/μL)	Lymphocyte (%)	Neutrophil (%)
Group 1: Deionised water (10 ml/kg)	40.67 ± 1.63	16.31 ± 0.72	7.89 ± 0.18	19.24 ± 4.85	71.00 ± 3.35	26.83 ± 3.55
Group 2: Lead acetate (60 mg/kg)	32.83 ± 1.29^a^	12.57 ± 0.66^a^	4.41 ± 1.05^a^	30.66 ± 3.40^a^	77.00 ± 6.42	26.33 ± 1.51
Group 3: Lead acetate (60 mg/kg) + AC (1000 mg/kg)	38.75 ± 0.42^b^	15.47 ± 0.42^b^	7.45 ± 0.52^b^	18.12 ± 4.70^b^	69.00 ± 559	26.83 ± 2.93

*Data were expressed as mean ± SD. ^a^Significantly different when compared to the control group (*p* < 0.05). ^b^Significantly different when compared to the lead acetate-treated group (*p* < 0.05) (*n* = 6).*

### Effect of Activated Charcoal on Biochemical Parameters

**Table 2** show the effect of activated charcoal on the serum levels of AST, ALP, and ALT in lead acetate-treated male albino Wistar rats. Treatment of rats with lead acetate (group 2) caused significant (*p* < 0.05) increase in the following liver enzymes when compared with the normal control group (group 1): AST, ALP, and ALT. These enzymes decreased significantly (*p* < 0.05) in group 3 animals which were given activated charcoal after lead acetate treatment (**Table 2**).

**Table 2:** Effect of activated charcoal on the serum levels of Aspartate aminotransferase (AST), Alkaline phosphatase (ALP) and Alanine aminotransferase (ALT) in Lead acetate-treated male albino Wistar rats.

Treatment	AST (U/L) (U/L)	ALP (U/L)	ALT (U/L)
Group 1: Deionised water (10 ml/kg)	55.77 ± 0.88	180.79 ± 0.17	17.67 ± 2.05
Group 2: Lead acetate (60 mg/kg)	76.77 ± 9.20^a^	211.71 ± 9.63^a^	35.53 ± 2.82^a^
Group 3: Lead acetate (60 mg/kg) + AC (1000 mg/kg)	53.82 ± 5.93^b^	183.31 ± 15.07^b^	19.39 ± 5.00^b^

*Data were expressed as mean ± SD. ^a^Significantly different when compared to the control group (*P* < 0.05). ^b^Significantly different when compared to the lead acetate-treated group (*P* < 0.05) (*n* = 6).*

The effect of activated charcoal on serum total proteins, Albumins, Globulins, Urea, Creatinine, and Bilirubin in lead acetate-treated male albino Wistar rats is shown on **Table 3**. There was significant (*p* < 0.05) decrease in serum total proteins and albumins in rats in group 2 compared to rats in group 1. These two parameters increased significantly (*p* < 0.05) in group 3 animals given activated charcoal after lead acetate treatment. The serum level of globulin was not significantly affected (**Table 3**). There was a significant increase (*p* < 0.05) in the following parameters: urea and bilirubin in group 2 animals compared to that of rats in the normal control group (group 1). These parameters decreased significantly (*p* < 0.05) in group 3 animals. However, there was no significant effect (*p* > 0.05) on creatinine level.

**Table 3:** Effect of activated charcoal on serum total proteins, albumins, globulins, urea, creatinine, and bilirubin in lead acetate-treated male albino Wistar rats.

Treatment	Total proteins (g/dl)	Albumins (g/dl)	Globulins (g/dl)	Urea (mg/dl)	Creatinine (mg/dl)	Bilirubin (mg/dl)
Group 1: Deionised water (10 ml/kg)	8.55 ± 0.34	4.28 ± 0.38	4.27 ± 0.35	17.92 ± 2.83	0.59 ± 0.08	0.27 ± 0.11
Group 2: Lead acetate (60 mg/kg)	6.61 ± 0.16^a^	2.90 ± 0.17^a^	3.71 ± 0.25	29.00 ± 3.23^a^	0.64 ± 0.05	0.97 ± 0.31^a^
Group 3: Lead acetate (60 mg/kg) + AC (1000 mg/kg)	7.76 ± 0.32^ab^	3.90 ± 0.14^b^	3.84 ± 0.38	18.91 ± 1.47^b^	0.56 ± 0.05	0.35 ± 0.10^b^

*Data were expressed as mean ± SD. ^a^Significantly different when compared to the control group (*P* < 0.05). ^b^Significantly different when compared to the lead acetate-treated group (*p* < 0.05) (*n* = 6).*

On the lipid profiles, rats in group 2 showed significant (*p* < 0.05) increase in total cholesterol, triglycerides, LDL and VLDL, compared to those in the control group. These parameters were significantly (*p* < 0.05) reduced in group 3 animals except for VLDL. There was no significant effect (*p* > 0.05) on HDL values (**Table 4**).

**Table 4:** Effect of activated charcoal on lipid profiles of lead acetate-treated male albino Wistar rats.

Treatment	Total cholesterol (mg/dl)	HDL (mg/dl)	LDL (mg/dl)	VLDL (mg/dl)	Triglyceride (mg/dl)
Group 1: Deionised water (10 ml/kg)	53.29 ± 5.57	22.16 ± 6.22	26.30 ± 9.21	4.94 ± 0.47	24.17 ± 2.84
Group 2: Lead acetate (60 mg/kg)	78.51 ± 13.66^a^	22.62 ± 5.00	40.07 ± 4.92^a^	22.64 ± 12.64^a^	113.20 ± 63.17^a^
Group 3: Lead acetate (60 mg/kg) + AC (1000 mg/kg)	55.03 ± 5.74^b^	20.56 ± 3.30	26.06 ± 1.51^b^	10.69 ± 4.55	45.70 ± 20.39^b^

*Data were expressed as mean ± SD. ^a^Significantly different when compared to the control group (*P* < 0.05). ^a^Significantly different when compared to the lead acetate-treated group (*P* < 0.05) (*n* = 6).*

**Table 5** shows the effect of activated charcoal on some antioxidant, lipid peroxidation parameters and pro-inflammatory cytokines of Lead acetate-treated male albino Wistar rats.

**Table 5:** Effect of activated charcoal on some antioxidant, lipid peroxidation parameters and pro-inflammatory cytokines of lead acetate-treated male albino Wistar rats.

Treatment	Malondialdehyde (μmol/L of plasma)	Glutathione peroxidase (U/L of blood)	Superoxide dismutase (U/ml of blood)	Total glutathione (ng/μL)	Interleukin-6 (IL-6)	Tumor necrosis factor-alpha (TNF-α)
Group 1: Deionised water (10 ml/kg)	1.58 ± 0.09	482.85 ± 53.43	144.80 ± 7.00	1.11 ± 0.03	74.41 ± 5.45	0.01 ± 0.00
Group 2: Lead acetate (60 mg/kg)	1.90 ± 0.17^a^	247.18 ± 70.40^a^	122.39 ± 4.63^a^	0.56 ± 0.31^a^	113.58 ± 13.46^a^	0.00 ± 0.00
Group 3: Lead acetate (60 mg/kg) + AC (1000 mg/kg)	1.62 ± 0.14^b^	327.65 ± 96.32	142.13 ± 8.82^b^	1.20 ± 0.08^b^	72.68+11.68^b^	0.00 ± 0.00

*Data were expressed as mean ± SD. ^a^Significantly different when compared to the control group (*P* < 0.05). ^b^Significantly different when compared to the lead acetate-treated group (*P* < 0.05) (*n* = 6).*

Treatment of rats with lead acetate (group 2) caused significant (*p* < 0.05) decrease in the following antioxidant enzymes/parameters: GSH-PX, SOD, and Total Glutathione when compared with the normal control group (group 1). These parameters were significantly (*p* < 0.05) increased in group 3 animals. Also, the value of the biomarker of lipid peroxidation, MDA was significantly (*p* < 0.05) increased in group 2 rats, compared to those in the normal control group (group 1). This biomarker was significantly (*p* < 0.05) decreased in group 3. Rats in group 2 showed significant (*p* < 0.05) increase in the pro-inflammatory cytokine, IL-6 in comparison with normal control (group 1). Level of this cytokine was significantly (*p* < 0.05) decreased in group 3. However, there was no effect on the serum level of another pro-inflammatory cytokine, TNF-α.

### Histopathology of the Kidney and liver:

#### Liver

**FIGURE 1:**
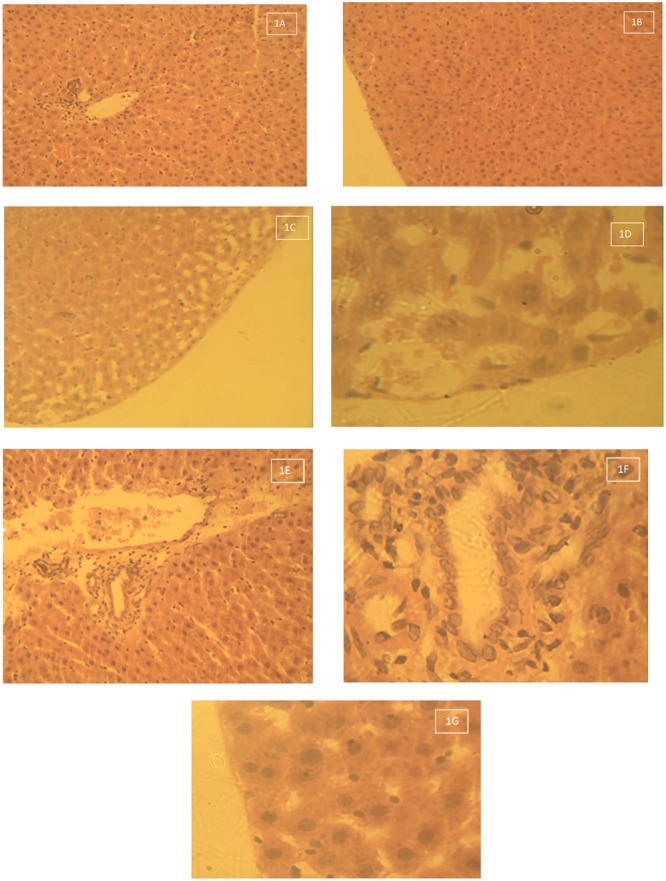
**(A)** Histological photomicrograph of the liver tissue stained with H&E techniques of Group 1 – control that received deionised water 10 ml/kg by oral gavage daily for 28 days – Central veins with normal intact hepatocytes surrounding it (1A Mag × 160). **(B)** Histological photomicrograph of the liver tissue stained with H&E techniques of Group 1 – control that received deionised water 10 ml/kg by oral gavage daily for 28 days – Intact hepatocytes on the periphery (no vacuolations – 1B Mag × 640). **(C)** Histological photomicrograph of the liver tissue stained with H&E techniques of Group 2 – Lead acetate 60 mg/kg daily for 28 days – Massive cellular infiltration around the portal areas with some hepatocyte necrosis (1C Mag × 160). **(D)** Histological photomicrograph of the liver tissue stained with H&E techniques of Group 2 – Lead acetate 60 mg/kg daily for 28 days – Vacuolation or vacuolar degeneration of peripheral hepatocytes (1D Mag × 640). **(E)** Histological photomicrograph of the liver tissue stained with H&E techniques of Group 3 – Lead acetate (60 mg/kg) followed by AC (1000 mg/kg) daily for 28 days – Mild cellular infiltration around the portal areas (1E Mag × 160). **(F)** Histological photomicrograph of the liver stained with H&E techniques of Group 3 – Lead acetate (60 mg/kg) followed by AC (1000 mg/kg) daily for 28 days – Mild cellular infiltration with few lymphocytes around the portal areas (1F Mag × 640). **(G)** Histological photomicrograph of the liver stained with H&E techniques of Group 3 – Lead acetate (60 mg/kg) followed by AC (1000 mg/kg) daily for 28 days – Intact hepatocyte.

The histological photomicrograph of the liver tissue stained with H&E techniques of Group 1 – control that received deionised water 10 ml/kg by oral gavage daily for 28 days is shown on **Figures 1A,B**. Central veins with normal intact hepatocytes surrounding it (1A Mag × 160). **Figures 1C,D** show the histological photomicrograph of the liver tissue stained with H&E techniques of Group 2 – Lead acetate 60 mg/kg daily for 28 days. Liver samples from group 2 (lead acetate-treated group) were observed to have massive cellular (neutrophils and lymphocytes) infiltrations around the peri-portal areas, some hepatocyte necrosis and there was also vacuolations/vacuolar degeneration of peripheral hepatocytes (**Figures 1C,D**). **Figures 1E,F,G** show the Histological photomicrograph of the liver tissue stained with H&E techniques of Group 3 – Lead acetate (60 mg/kg) followed by AC (1000 mg/kg) daily for 28 days. There was no vacuolar degeneration of peripheral hepatocytes (intact hepatocytes) and mild cellular infiltration with few lymphocytes around the portal areas

#### Kidney

**FIGURE 2:**
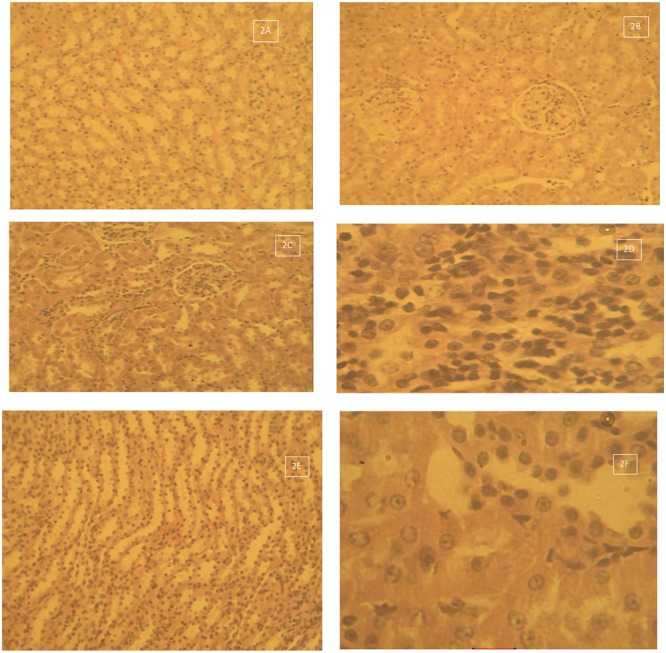
**(A)** Histological photomicrograph of the kidney stained with H&E techniques of Group 1 – control that received deionised water 10 ml/kg by oral gavage daily for 28 days – normal dilated tubules and glomeruli at the periphery of the cortex (2A Mag × 160). **(B)** Histological photomicrograph of the kidney stained with H&E techniques of Group 1 – control that received deionised water 10 ml/kg by oral gavage daily for 28 days – normal renal cortical tubules and glomeruli (2B Mag × 640). **(C)** Histological photomicrograph of the kidney tissue stained with H&E techniques of Group 2 – Lead acetate 60 mg/kg daily for 28 days – Degeneration and necrosis of the renal parenchymal cells with massive inflammatory cells infiltration (2C Mag × 160). **(D)** Histological photomicrograph of the kidney tissue stained with H&E techniques of Group 2 – Lead acetate 60 mg/kg daily for 28 days – Massive lymphocytic cellular infiltration, with degeneration and necrosis (2D Mag × 640). **(E)** Histological photomicrograph of the kidney stained with H&E techniques of Group 3 – Lead acetate (60 mg/kg) followed by AC (1000 mg/kg) daily for 28 days –showing normal medullary tubules (2E Mag × 160). **(F)** Histological photomicrograph of the kidney stained with H&E techniques of Group 3 – Lead acetate (60 mg/kg) followed by AC (1000 mg/kg) daily for 28 days – some degenerated tubules and mild cellular infiltration of the renal parenchyma(2F Mag × 640).

**Figure 2** shows the histological photomicrograph of the kidney stained with H&E techniques of Group 1 – control that received deionised water 10 ml/kg by oral gavage daily for 28 days (**Figures 2A,B**). There was normal dilated tubules and glomeruli at the periphery of the cortex. Group 2 – Lead acetate 60 mg/kg daily for 28 days –Degeneration and necrosis of the renal parenchymal cells with massive inflammatory cells infiltration (2C Mag × 160). The kidney of rats in Group 2 – Lead acetate 60 mg/kg daily for 28 days showed areas of degeneration and necrosis of the renal cortical parenchymal cells with massive lymphocytic cellular infiltration, with vacuolation/vacuolar degeneration of the peripheral interstitial cells of the cortex (**Figures 2C,D**). Tissue sections from the kidneys of rats in Group 3 – Lead acetate (60 mg/kg) followed by AC (1000 mg/kg) daily for 28 days had varied areas of normal and degenerated cortical tubules, with mild cellular infiltration of the renal parenchyma (**Figures 2E,F**).

## Discussion

Lead is one of the most toxic heavy metals of great public health significance. Exposure to low-level heavy metals such as lead may contribute much more toward the causation of chronic diseases (diabetes, renal disease, cancer, male infertility etc.) and impaired functioning than previously thought (Orisakwe, 2014). Chelators, the currently available antidote for the treatment of lead poisoning have many adverse effects such as being painful, hepatotoxicity, gastrointestinal symptoms, among others. In addition to difficulties in the administration of some chelators, they are also expensive and not readily available. Also, chelation therapy is recommended by the CDC to be used only in extreme cases where the blood lead level is over 45 ug/dL. It is generally not used in cases where levels are under 25 ug/dl and it neither removes all of the lead from the body nor does it undo damage already done to organ. Activated charcoal is thus investigated in this study as a possible alternative to the classical antidotes owing to the aforementioned limitations. As a further justification for this research, there is currently sparse information on the *in vivo* efficacy of AC in the treatment of lead poisoning.

In this study, treatment of rats with lead acetate caused significant increase in the activity of serum AST, ALT, ALP, bilirubin and urea, while the levels of albumin and total proteins were decreased. Similar results were reported by Azoz and Raafat (2012), Ibrahim et al. (2012), and Azab (2014). These parameters were, however, reversed by treatment with activated charcoal in this study. Increasing levels of AST and ALT in the lead acetate-treated rats signify damage to the structural integrity of the liver. It is mainly due to the leakage of these enzymes from the liver cytosol into the blood stream (Concepcion et al., 1993). Releasing of AST and AST from the cell cytosol can occur as secondary changes to cellular necrosis (Gaskill et al., 2005). The high AST and ALT activities are accompanied by high liver microsomal membrane fluidity, free radical generation and alteration in the liver tissue (Ibrahim et al., 2012). Elevated level of ALP suggests biliary damage or an obstruction of the biliary tree, which disrupts the flow of blood to the liver (Farida et al., 2012). The decrease in serum levels of these enzymes may be due to the prevention of their leakage from the liver cytosol by activated charcoal, probably due to reduction in blood lead level.

The increase of bilirubin values in rats treated with lead acetate in this study may be due to excessive heme destruction and blockage of biliary tract resulting in inhibition of the conjugation reaction and release of unconjugated bilirubin from damaged hepatocytes (Ali et al., 2010). Bilirubin has a protective role against oxidative damage of cell membrane induced by metal (Noriega et al., 2003).

Lead acetate administration in this study caused significant increase in serum urea level, but only a slight increase in serum creatinine level. The serum urea was significantly decreased with the administration of activated charcoal in this study but showed only a slight decrease in the value of creatinine after 28 days. This is in agreement with the work of Cheong and Roh (2006), whose data showed a significant decrease in the value of blood urea nitrogen after 1 week of administration of activated charcoal, while the serum level of creatinine was only significantly reduced by activated charcoal after 72 h. This study confirms the conclusion by Cheong and Roh (2006) that activated charcoal may protect the lead-induced toxicity on kidney.

Charcoal in various forms administered with low protein diets has been reported to control effectively some uremic symptoms in patients with different stages of renal disease, and this is achieved through the binding of urea and other urinary toxins to charcoal, and its excretion with feces, creating a concentration gradient for continued diffusion of these toxins (Ash, 2009). Simultaneous treatment of rats with adenine and AC (20% w/w in the feeds for 28 days) has been shown to produce a broad, dose-dependent, nephroprotective action in adenine-induced chronic Renal failure (Ali et al., 2014). Also in this study, lead acetate caused a significant decrease in the values of serum total proteins and Albumins. Administration of activated charcoal in this study was able to significantly increase these parameters. Decrease in serum total protein may be due to both hepatic and renal damage induced by lead (Ahmed and Shalaby, 1999), or may be due to binding of lead to plasma proteins, where it causes alteration in a high number of enzymes and can also disturb protein synthesis in hepatocytes (Goering, 1993). Decreasing of serum total protein values may be attributed to a decrease in hepatic DNA and RNA induced by lead intoxication or due to decreased utilization of free amino acids for protein synthesis (Moussa and Bashandy, 2008).

In our study, administration of lead acetate was found to cause elevation of Total cholesterol, triglycerides, LDL and VLDL. These parameters were reduced by treatment with activated charcoal. The result is in agreement with the report of Azoz and Raafat (2012). The high lipid levels could be due to either increased synthesis or decreased removal of lipoproteins. Decreased removal may occur as a result of the alteration of cell – surface receptors for lipoprotein (Tarugi et al., 1982) or as a result of the inhibition of hepatic lipoprotein lipase activity (Chajet et al., 1989). Furthermore, lead has been shown to depress the activity of cytochrome P – 450 (Alvares et al., 1975), this can limit the biosynthesis of the bile acids, which is the significant route for elimination of cholesterol from the body. Although activated charcoal did not show any significant effect on HDL in this study, AC significantly lowered lead induced increase in LDL, triglycerides and total cholesterol implicated in the development of heart disease.

It was observed in this study that levels of total glutathione, SOD, GSH-PX were significantly reduced in lead acetate-treated rats. The values of these biomarkers were increased by the protective activity of activated charcoal, which also significantly reduced the value of the lead acetate-induced biomarker of lipid peroxidation, MDA. This result is in agreement with the report of Azoz and Raafat (2012). Oxidative stress represents an imbalance between the production of free radicals and the biological system’s ability to readily detoxify the reactive intermediates or to repair the resulting damage (Flora, 2011). It has been reported as a major mechanism of lead induced toxicity (Flora et al., 2012). Under the influence of lead, onset of oxidative stress occurs on account of two different pathways operative simultaneously. First, the generation of reactive oxygen species, ROS and second, the antioxidant reserves become depleted (Flora, 2002). Apart from targeting the sulfhydryl groups, lead can also replace the zinc ions that serve as important cofactors for these antioxidant enzymes and inactivate them (Flora et al., 2007). Lipid peroxidation, another indicator of oxidative stress occurs as a result of the action of ROS on lipid membranes. The generated free radical captures electrons from the lipids present inside the cell membranes and damages the cell.

Treatment of rats with lead acetate in this study caused significant reduction in PCV, Hb concentration and RBC count. Our results were in agreement with Azoz and Raafat (2012) and Ibrahim et al. (2012). On the other hand, total WBC was significantly increased. Administration of activated charcoal significantly reversed these parameters. Lead directly affects the hematopoietic system through restraining the synthesis of Hb by inhibiting various key enzymes involved in the heme synthesis pathway, particularly the enzyme Aminolevulinic Acid Dehydratase (ALAD)_._ It also reduces the life span of circulating erythrocytes by increasing the fragility of cell membranes. The aftermath of these two processes leads to anemia (Guidotti et al., 2008; Flora et al., 2012).

Administration of lead acetate caused significant increase in the pro-inflammatory cytokine, IL-6. However, administration of activated charcoal significantly reduced the level of IL-6. AC could protect from lead acetate-induced toxicity by attenuating the increased serum IL-6. However, the exact mechanism through which it is achieved requires further investigation. Howell et al. (2013), have reported good removal of the inflammatory cytokines IL-8 (100% removal), IL-6 (80% removal) and TNF-α (51% removal) from blood using nanoporous activated carbon beads. In another experiment, Inoue et al. (2015) concluded that AC should be efficient for cytokine adsorption. Measurements of humoural factors such as cytokines and other inflammatory mediators or markers can provide predictive clinical information plus insights into disease mechanisms (Zhou et al., 2010).

Taken together activated charcoal seems to be protective against hepato-renal damage induced by chronic exposure of lead in the animal model. The elevation of antioxidant enzymes level, reduction of lipid peroxidation (MDA), modulation of the pro-inflammatory cytokine, IL-6; and reversal of lead-induced alteration in some hematological and biochemical parameters following administration of AC may be as result of decrease in blood lead level BLL, Further studies may be necessary to understand the precise mechanism of action.

## Author Contributions

SO performed bench study, write up and analyses of data. HM designed study and OO conceptualized, designed the study and write up.

## Conflict of Interest Statement

The authors declare that the research was conducted in the absence of any commercial or financial relationships that could be construed as a potential conflict of interest.
